# Impacts of biopolymer infusion on quality traits of pork sausages during storage

**DOI:** 10.5713/ab.24.0743

**Published:** 2025-02-27

**Authors:** Sang-keun Jin, Donggyun Yim

**Affiliations:** 1 Division of Animal Science, Gyeongsang National University, Jinju, Korea; 2 Department of Animal Science and Biotechnology, Kyungpook National University, Sangju, Korea; 3 Research Institute for Innovative Animal Science, Kyungpook National University, Sangju, Korea

**Keywords:** Biopolymer, Cellulose, Chitosan, Sausage Quality, Storage

## Abstract

**Objective:**

We aimed to verify the impacts of cellulose/chitosan addition on the physicochemical properties of sausages.

**Methods:**

Sausages were prepared using 3% cellulose or chitosan and compared with those without biopolymers (control).

**Results:**

Sausages containing chitosan exhibited decreased pH values (p< 0.05), whereas those containing cellulose exhibited increased pH values. 2-thiobarbituric acid reactive substances values did not differ between the control and biopolymer samples. The biopolymer samples had lower volatile basic nitrogen contents than the control samples after 4 weeks of storage (p<0.05). The addition of chitosan significantly reduced the microbiological counts of sausages. Moreover, the infusion of chitosan led to lower lightness (L*) and whiteness (W) values but higher redness (a*), yellowness (b*), chroma (C*), and hue-angle (*h*°) values (p<0.05). During storage, the sausages containing chitosan exhibited significantly better textural parameters, except for springiness and gumminess.

**Conclusion:**

In conclusion, adding chitosan could reduce microbial growth and prolong the expiration date of sausages without compromising quality.

## INTRODUCTION

Sausages are a meat-based product primarily comprising meat and varying levels of fat. The meat and fat are diced and blended with ice, along with a selection of additives. After thorough mixing, the mixture is filled into casings and refrigerated until consumption. Given their elevated fat content and the absence of heat treatment, sausages are susceptible to spoilage resulting from both lipid peroxidation and microbial contamination [1,2].

Chitosan, the second most prevalent biopolymer following cellulose and a derivative of chitin, has attracted attention for its various health benefits, including antimicrobial, antioxidant, and hypocholesterolemic effects [3]. As this polysaccharide is discovered in the shells of crustaceans, it is harmless, environmentally degradable, and biocompatible [4]. Consequently, it has been approved as “generally recognized as safe” (GRAS) [5]. Moreover, the substantial quantity of by-products and waste generated by the marine food processing industry serves as a recyclable and eco-friendly reservoir of chitosan. This not only aids in minimizing environmental issues caused by these residues but also facilitates their effective utilization [4]. Furthermore, chitosan exhibits emulsifying, water retention, and lipid retention properties, improves cooking yield, and adds color [6]. Incorporating chitosan into sausage formulations can enhance meat quality and extend shelf life while addressing the health concerns associated with processed meats. Chitosan has been found to exhibit antioxidant and preservative effects in sausages [7–11]. Moreover, the incorporation of chitosan has been reported to have minimal detrimental impacts on the quality traits of sausages [7,10].

Cellulose, the most abundant biopolymer in plants, is the major polysaccharide in the plant cell wall [12]. As it contains polysaccharides, it is generally considered safe for consumption in reasonable amounts. In addition to its stabilizing properties, cellulose can be used to increase lightness in food [13]. Some research has been conducted on the quality traits of bacterial cellulose in sausages [14–16]. Amorphous cellulose is not a soluble fiber and has no taste or calories [17]. The addition of amorphous cellulose has been reported to be an alternative for pork back fat in sausages [1,2]. In contrast, little research has been carried out on the direct addition of simple cellulose itself instead of bacterial or amorphous cellulose into the formulation of cooked pork sausage. Further research on the impact of reformulating meat products through the incorporation of cellulose or chitosan is crucial for understanding the technological and industrial implications. However, to date, very limited information is available on the use of biopolymers, such as cellulose or chitosan, as additives in sausages. Hence, we aimed to identify the effects of cellulose or chitosan on the qualitative traits of sausages and their stability during storage.

## MATERIALS AND METHODS

### Sausage preparation

Three types of samples were prepared; the compositions are detailed in Table 1. The sausages were prepared thrice, with 12 kg batches independently prepared for each treatment. Fresh pork ham and back fat were ground into particles measuring 4 mm in size. The composition of each batch was as follows: 72.44% lean pork, 11.2% back fat, 13.8% ice, 0.01% sodium nitrite, 0.2% sodium triphosphate, 0.5% sugar, 0.05% monosodium glutamate, and 0.4% spices. Compared with the group without biopolymers (control), the experimental groups involving biopolymers were treated with 3% cellulose based on the findings of preliminary studies. The formulation was determined through preliminary assessments of different biopolymer concentrations ranging from 1% to 5%. The mixture of all ingredients was blended using a low-speed mixer (Fujee Co., Seoul, Korea) for 15 min. Emulsification was carried out at high speed of 11,500×g for about 7 min while ensuring that the mixture temperature remained below 15°C. The resulting batters were subsequently stuffed into fibrous casings with a diameter of 60 mm. The sausages were cooked in a smoke chamber (Thematec Food Industry Co., Seoul, Korea) until the core temperature reached 75°C to 78°C. Then, the sausages were cooled to 4°C in a refrigerator and stored for 4 weeks.

### Physico-chemical analysis

pH measurements were conducted with a pH meter (MP330; Mettler Toledo, Greifensee, Switzerland) using 3 g of sausage products homogenized in a 1:9 ratio with distilled water. Salinity was determined according to the method reported by Chen et al [18]. In brief, a 5 g sample was homogenized with 45 mL of distilled water, filtered through Whatman No. 1 filter paper, and then subjected to salinity analysis. Purge loss was determined by assessing the weight difference of sausages between the end of 2 and 4 weeks of storage in vacuum bags with their initial weight. Thiobarbituric acid reactive substance (TBARS) values were evaluated using the procedure outlined by Jeong et al [19]. In brief, 5 g of the sample was mixed with 45 mL of distilled water and 50 μL of butylated hydroxyanisole. The blend was then agitated for 1 min at the highest speed. Subsequently, 1 mL of the resulting blend was combined with 2 mL of TBA solution (consisting of 0.25 N HCl, 15% TCA, and 0.375% TBA reagent) and heated at 90°C for 15 min. After centrifugation at 3,100×g and 4°C for 9 min, readings were obtained at 531 nm using an X-MA 4000 spectrophotometer (Human Co. Ltd., Seoul, Korea). TBARS values were calculated as absorbance (O.D.)×7.8 (mg of malondialdehyde/kg of sample). Volatile basic nitrogen (VBN) contents were determined using Conway’s method [19]. Meat color was assessed using a chroma meter (CR-400; Minolta Co., Osaka, Japan). Total aerobic bacteria (TAB) were assessed by plating the diluted homogenate onto TAB agar and incubating at 37°C for 24 h. Colony counts were expressed as log CFU/g. Meat texture was evaluated using a texture analyzer (TA-XT4; Stable Micro Systems, Surrey, UK). Various parameters, including hardness, springiness, cohesiveness, gumminess, chewiness, adhesiveness, and shear forces, were measured on sliced samples (19 mm diameter, 10 mm thickness) at a crosshead speed of 6 mm/s.

### Statistical analysis

The experiment was performed at different times in the same location, and a completely randomized design was used. Analysis based on the general linear model was conducted using SAS 8.3 software (SAS Institute Inc., Cary, NC, USA). Data were reported as mean values and standard errors of the means. The significance of differences among mean values was determined using Duncan’s multiple range test; the significance level was set at p<0.05.

## RESULTS AND DISCUSSION

Table 2 presents the quality characteristics of sausages manufactured using either chitosan or cellulose during storage. Regardless of storage, the control and cellulose sausages exhibited higher pH values than the chitosan samples (p<0.05). The cellulose samples exhibited the highest pH values (p<0.05). Similar data were reported by Qin et al [10]; they found that the chitosan groups exhibited lower pH values than the control group. The decline in pH may be owing to microbial changes because the growth of acid-producing bacteria may lower the pH [20]. This indicates that microbial control by the insertion of chitosan can help lower the pH. On the other hand, in some studies [5,6], chitosan groups exhibited higher pH values than the control group. The increase in pH is attributed to the alkaline properties of chitosan, which are enhanced by the presence of amino groups [21]. As cellulose is a nonionic polymer, it remains unaffected by fluctuations in pH because of its lack of charge [22]. Regardless of biopolymer addition, the control samples had a similar salinity content to the biopolymer samples. Purge loss was higher in the chitosan sausages than in the control and cellulose samples at 2 and 4 weeks (p<0.05). Purge loss in the chitosan samples increased until 2 weeks and then decreased for 4 weeks (p<0.05).

The TBARS value of the control sausages was similar to that of the biopolymer samples (p>0.05). The sausages containing chitosan and cellulose found no significant increase in TBARS values throughout the storage duration. This suggests that the presence of chitosan and cellulose effectively prevents the development of lipid oxidation during storage. The addition of cellulose as a stabilizer in meat products can enhance the oxidation stability of emulsions [23]. Han et al [24] noted no significant differences between meat samples containing cellulose and control samples. The effects of chitosan in controlling lipid oxidation in sausages have been documented in numerous studies [5,21]. However, the results suggested that both samples containing chitosan and control samples exhibit comparable values during storage. TBARS values below 1 mg MDA/kg are linked to negligible or barely noticeable alterations in oxidation in meat and meat products [25]. In our study, TBARS values remained below 1 mg MDA/kg throughout storage, irrespective of treatments. Thereafter, the incorporation of 3% cellulose or chitosan can effectively inhibit lipid oxidation in samples. The biopolymer samples had a lower VBN content than the control samples after 4 weeks of storage (p< 0.05). The reduced VBN contents in the sausages upon the incorporation of biopolymers could be attributed to a rapid decline in microbial counts and a decrease in the ability of bacteria to undergo oxidation-induced breakdown of nonprotein nitrogen compounds [17]. Consequently, the incorporation of cellulose or chitosan biopolymers had a beneficial impact on VBN contents in sausage samples during the storage period.

As shown in Table 2, chitosan incorporation induced a decrease in total microbial counts in sausages; this difference was maintained throughout the storage duration. The total microbial count in the chitosan samples (3.71 log_10_ CFU/g) was lower than those in the control and cellulose samples (5.79 and 5.57 log_10_ CFU/g, respectively) at 4 weeks (p<0.05). Similar counts have been reported by many researchers [5,6,7,10], indicating a 2 log unit decrease in total microbial counts on the addition of chitosan. All samples exhibited a significant increase in the total viable counts gradually (p<0.05) over the storage duration. After 4 weeks of storage, the total counts in the control and cellulose samples reached up to 5 log CFU/g. However, the total counts in the chitosan samples were approximately 2 log CFU/g lower than those in the control and cellulose samples. These findings are consistent with previous findings [5,20,21] that chitosan incorporation enhanced the shelf life of sausages. Various mechanisms have been suggested to clarify the antimicrobial properties of chitosan. Chitosan can sequester nutrients required by bacteria, thereby hindering their growth [26]. Moreover, the interaction between positively charged chitosan molecules and negatively charged microbial cell membranes leads to the release of intracellular contents, including proteins [18]. Furthermore, chitosan functions as a chelating agent, selectively binding trace metals and consequently inhibiting the synthesis of toxins and the proliferation of microorganisms [27]. A decline in pH facilitates the protonation of chitosan, thereby amplifying its antimicrobial efficacy [3]. The reduced microbial count found in the chitosan samples may be owing to the antimicrobial properties of chitosan powder. The antimicrobial action of chitosan is thought to stem from the electrostatic attraction between the positively charged NH3^+^ group of glucosamine monomers within chitosan molecules and the negatively charged microbial cell membrane, resulting in the leakage of intracellular components [28]. This finding suggests that the addition of chitosan can effectively restrain microbial growth in sausages. Thus, chitosan may serve as a natural antimicrobial agent in sausages.

Table 3 indicates the colors of sausages incorporated with cellulose or chitosan during storage. The L* values of sausages containing cellulose were higher, while those of sausages containing chitosan were lower (p<0.05). The bright white color of cellulose may have contributed to the increase in L* values upon addition to the sausages [29]. In contrast, the decrease in L* values in the chitosan samples may be attributed to the water-holding capacity of chitosan [30], suggesting that an increase in ater holding capacit reduces the L* value. This seems to be consistent with the findings reported by Sayas-Barberá et al [6]. In their study, the L* value of samples increased during storage. This increase may be attributed to the extent of oxidation and level of metmyoglobin.

The redness value serves as a particularly pertinent indicator of alterations in the red hue of meat products. The cellulose samples showed lower redness values than the control and chitosan samples following 2 and 4 weeks of storage (p< 0.05). Similar trends were noted in the redness values between the chitosan and control samples during storage. Lee et al [31] examined the stability of pork meat infused with chitosan and found that the meat color, as represented by the redness value, remained consistent throughout storage. This preservation of the red color of meat can be attributed to the chelating abilities of chitosan. These findings confirm that chitosan acts as a natural antioxidant exhibiting a similar effect to nitrite in preserving the red color. In a previous study, 1% chitosan effectively prevented surface discoloration of refrigerated ground steak by maintaining lower redness values and higher redness values in case-ready packaging [32].

The b* values were the highest in the chitosan samples (p< 0.05), wherein chitosan affected the yellow color of the sausages. This seems consistent with previous findings [6,20,33] that adding chitosan increased yellowness, indicating that the inherent color of chitosan influences surface coloration. Regardless of storage, the chitosan samples had a lower *W* value than the control and cellulose samples; however, the chitosan samples had a higher saturation index (C*) and hue (*h*) value (p<0.05).

Table 4 presents the textural properties of sausages containing cellulose or chitosan during storage. Regardless of storage, the chitosan samples had higher hardness, cohesiveness, chewiness, adhesiveness, and shear force values, whereas the control samples had lower values (p*<*0.05). These findings indicate that hardness increased with the addition of chitosan. Similar to our results, previous studies [24,34,35] reported higher hardness values in the chitosan samples than in the control samples. This occurrence could be attributed to the binding characteristics of chitosan, facilitating the creation of a more robust gel structure with myosin and water [35]. Moreover, this behavior may be attributed not only to the slight dehydration of the product during storage but also to the stabilization of chitosan linkages with matrix components under cold temperatures [36].

While adding chitosan leads to an increase in hardness, this outcome is considered favorable as it helps confer a more robust structure to sausages. Moreover, the effect of chitosan on chewiness was similar to that on hardness, consistent with the findings reported by Han et al [24]. Compared with the control samples, the chitosan samples exhibited slightly increased cohesiveness and chewiness. Chewiness, described as the product of hardness and cohesiveness, is influenced by hardness. Therefore, a parallel increase was noted in these parameters with the addition of chitosan during storage. The control sausages exhibited the lowest shear force values (p<0.05).

## CONCLUSION

This study demonstrated that incorporating chitosan effectively maintained the physicochemical properties of sausages. Specifically, chitosan showed potential for satisfying consumer preferences for meat products free from chemical preservatives and proved to be a beneficial additive for prolonging the shelf life of sausages. Lipid oxidation levels remained similar across all samples, indicating that the addition of chitosan did not affect TBARS values. Moreover, chitosan positively influenced the color of sausages. Additionally, we identified that chitosan could act as a natural antimicrobial agent in sausages. In conclusion, the incorporation of chitosan at a concentration of 3% not only extends the shelf life of sausages but also maintains lipid oxidation levels without compromising quality. Further research is needed to clarify the biological impacts of chitosan as a natural biopolymer in the meat industry.

## Figures and Tables

**Table 1:** Formulation of cooked sausage batter

Ingredient	Treatments (%)		

	Control	Cellulose	Chitosan
Pork lean meat	72.44	72.44	72.44
Pork back fat	11.2	11.2	11.2
Water (ice)	13.8	13.8	13.8
NaNO_2_	0.01	0.01	0.01
Phosphate	0.2	0.2	0.2
Sugar	0.5	0.5	0.5
MSG	0.05	0.05	0.05
Spices	0.4	0.4	0.4
Cellulose	-	3	-
Chitosan	-	-	3

MSG, sodium glutamate.

**Table 2:** Quality characteristics of sausages prepared with cellulose or chitosan during storage

Treatments	Storage			

	0 week	2 weeks	4 weeks	SEM
pH				
Control	6.46^Aa^	6.55^Aa^	6.08^Bb^	0.036
Cellulose	6.44^Aa^	6.47^Aa^	6.14^Ab^	0.025
Chitosan	6.37^Ba^	6.32^Ba^	6.00^Cb^	0.025
SEM^1)^	0.016	0.028	0.010	
Salinity (%)				
Control	2.00^a^	2.00^a^	1.40^b^	0.048
Cellulose	2.00^a^	2.00^a^	1.34^b^	0.051
Chitosan	2.00^a^	2.00^a^	1.48^b^	0.046
SEM	0.001	0.000	0.051	
Purge loss (%)				
Control	0.00^b^	1.49^Aa^	1.68^Aa^	0.117
Cellulose	0.00^b^	1.16^Ba^	1.04^Ba^	0.089
Chitosan	0.00^c^	0.93^Ba^	0.44^Cb^	0.067
SEM	0.000	0.069	0.093	
TBARS value (mg malonaldehyde/kg)				
Control	0.38^a^	0.32^b^	0.37^a^	0.005
Cellulose	0.35	0.35	0.36	0.009
Chitosan	0.35	0.31	0.38	0.018
SEM	0.058	0.064	0.061	
VBN content (mg%)				
Control	8.20^b^	8.37^Bb^	10.67^Aa^	0.219
Cellulose	8.41^b^	8.67^ABb^	9.42^Ba^	0.079
Chitosan	8.53^b^	8.74^Ab^	9.31^Ba^	0.073
SEM	0.059	0.065	0.174	
Total aerobic bacteria				
Control	0	4.14^Ab^	5.79^Aa^	0.348
Cellulose	0	3.25^Bb^	5.57^Aa^	0.341
Chitosan	0	0	3.71^Ba^	0.241
SEM	0	0.271	0.174	

1) n = 16.

a–c Numbers with different letters within the same row differ significantly (p<0.05).

A–C Numbers with different letters within the same column differ significantly (p<0.05).

SEM, standard error of the mean; TBARS, thiobarbituric acid reactive substances; VBN, volatile basic nitrogen.

**Table 3:** Color characteristics of sausages prepared with cellulose or chitosan during storage

Treatments	Storage			

	0 week	2 weeks	4 weeks	SEM
L* value				
Control	80.36^Ab^	81.05^Ba^	81.19^Ba^	0.082
Cellulose	80.60^Ab^	81.56^Aa^	81.64^Aa^	0.075
Chitosan	78.59^Bb^	79.03^Ca^	79.11^Ca^	0.059
SEM	0.136	0.159	0.158	
a* value				
Control	6.38^a^	5.81^ABb^	6.33^Aa^	0.063
Cellulose	6.21^a^	5.63^Bc^	5.91^Bb^	0.064
Chitosan	6.58^a^	5.93^Ac^	6.26^Ab^	0.051
SEM	0.070	0.047	0.036	
b* value				
Control	8.28^Bb^	8.61^Ba^	8.33^Bb^	0.043
Cellulose	8.28^Bb^	8.55^Ba^	8.33^Bab^	0.047
Chitosan	10.18^Ab^	10.69^Aa^	10.54^Aa^	0.050
SEM	0.130	0.142	0.149	
W value				
Control	55.59^A^	55.22^A^	56.18^A^	0.164
Cellulose	55.92^A^	55.91^A^	56.65^A^	0.153
Chitosan	48.12^Ba^	46.94^Bb^	47.51^Bab^	0.153
SEM	0.514	0.578	0.597	
C* value				
Control	10.47^B^	10.40^B^	10.47^B^	0.041
Cellulose	10.41^B^	10.24^B^	10.22^C^	0.044
Chitosan	12.11^A^	12.23^A^	12.26^A^	0.040
SEM	0.118	0.131	0.129	
h value				
Control	52.47^Bb^	56.01^Ba^	52.79^Cb^	0.353
Cellulose	53.29^Bb^	56.61^Ba^	54.63^Bb^	0.375
Chitosan	57.25^Ac^	60.98^Aa^	59.27^Ab^	0.270
SEM	0.456	0.373	0.422	

1) n = 16.

a–c Numbers with different letters within the same row differ significantly (p<0.05).

A–C Numbers with different letters within the same column differ significantly (p<0.05).

SEM, standard error of the mean; L, lightness, a, redness, b, yellowness, W, whiteness, C, chroma, h, hue.

**Table 4:** Textural properties of sausages prepared with cellulose or chitosan during storage

Treatments	Storage			

	0 week	2 weeks	4 weeks	SEM
Hardness (N)				
Control	0.19^Ca^	0.18^Cb^	0.20^Ca^	0.003
Cellulose	0.21^B^	0.21^B^	0.22^B^	0.002
Chitosan	0.24^Ab^	0.24^Ab^	0.26^Aa^	0.003
SEM^1)^	0.004	0.004	0.004	
Cohesiveness (g)				
Control	0.19^Ca^	0.18^Cb^	0.20^Ca^	0.003
Cellulose	0.21^B^	0.21^B^	0.22^B^	0.002
Chitosan	0.24^Ab^	0.24^Ab^	0.26^Aa^	0.003
SEM	0.004	0.004	0.004	
Springiness (g)				
Control	0.58^A^	0.59^AB^	0.59	0.004
Cellulose	0.56^Bb^	0.57^Bab^	0.58^a^	0.004
Chitosan	0.56^Bb^	0.60^Aa^	0.59^a^	0.005
SEM	0.004	0.004	0.004	
Gumminess (g)				
Control	1.00^ab^	1.00^b^	1.01^a^	0.001
Cellulose	1.00	1.00	1.00	0.001
Chitosan	1.00	1.00	1.01	0.004
SEM	0.001	0.001	0.003	
Chewiness (N)				
Control	0.11^Cb^	0.10^Cb^	0.12^Ca^	0.002
Cellulose	0.12^Bb^	0.12^Bb^	0.13^Ba^	0.002
Chitosan	0.13^Ac^	0.14^Ab^	0.15^Aa^	0.002
SEM	0.002	0.003	0.003	
Adhesiveness (g)				
Control	0.11^Cab^	0.10^Cb^	0.12^Ca^	0.002
Cellulose	0.12^Bb^	0.12^Bb^	0.13^Ba^	0.002
Chitosan	0.13^Ac^	0.14^Ab^	0.15^Aa^	0.002
SEM	0.002	0.003	0.003	
Shear force (kg/cm^2^)				
Control	0.11^Ba^	0.10^Cb^	0.11^Ca^	0.002
Cellulose	0.12^Aa^	0.11^Bc^	0.12^Bb^	0.001
Chitosan	0.13^Ab^	0.13^Ab^	0.14^Aa^	0.001
SEM	0.002	0.002	0.002	

1) n = 16.

a–c Numbers with different letters within the same row differ significantly (p<0.05).

A–C Numbers with different letters within the same column differ significantly (p<0.05).

SEM, standard error of the mean.
